# Regulation of skeletal muscle mitochondrial activity by thyroid hormones: focus on the “old” triiodothyronine and the “emerging” 3,5-diiodothyronine

**DOI:** 10.3389/fphys.2015.00237

**Published:** 2015-08-21

**Authors:** Assunta Lombardi, Maria Moreno, Pieter de Lange, Susanna Iossa, Rosa A. Busiello, Fernando Goglia

**Affiliations:** ^1^Department of Biology, University of Naples Federico IINaples, Italy; ^2^Department of Science and Technology, University of SannioBenevento, Italy; ^3^Department of Environmental, Biological and Pharmaceutical Sciences and Technologies, Second University of NaplesCaserta, Italy

**Keywords:** mitochondria, lipid metabolism, uncoupling, thyroid hormones, diiodothyronines

## Abstract

3,5,3′-Triiodo-L-thyronine (T3) plays a crucial role in regulating metabolic rate and fuel oxidation; however, the mechanisms by which it affects whole-body energy metabolism are still not completely understood. Skeletal muscle (SKM) plays a relevant role in energy metabolism and responds to thyroid state by remodeling the metabolic characteristics and cytoarchitecture of myocytes. These processes are coordinated with changes in mitochondrial content, bioenergetics, substrate oxidation rate, and oxidative phosphorylation efficiency. Recent data indicate that “emerging” iodothyronines have biological activity. Among these, 3,5-diiodo-L-thyronine (T2) affects energy metabolism, SKM substrate utilization, and mitochondrial functionality. The effects it exerts on SKM mitochondria involve more aspects of mitochondrial bioenergetics; among these, respiratory chain activity, mitochondrial thermogenesis, and lipid-handling are stimulated rapidly. This mini review focuses on signaling and biochemical pathways activated by T3 and T2 in SKM that influence the above processes. These novel aspects of thyroid physiology could reveal new perspectives for understanding the involvement of SKM mitochondria in hypo- and hyper-thyroidism.

## Introduction

Skeletal muscle (SKM) is a metabolically active tissue representing about 40% of total body mass. It significantly affects energy expenditure and plays a significant role in glucose, lipid, and energy homeostasis. SKM shows a remarkable plasticity in functional adaptation and remodeling in response to physiological stimuli, such as exercise, fasting, and hormonal signals. Among the hormones able to influence SKM development, metabolism, and structure, thyroid hormone (T3) plays a key role (Salvatore et al., 2014). SKM responds to variations in thyroid state by coordinately remodeling its cytoarchitecture and metabolic characteristics, with mitochondria playing a significant role. Concerning metabolic adaptations, T3 enhances the use of lipids and carbohydrates as fuel substrates (de Lange et al., 2008; Lombardi et al., 2012), as well as alteration of mitochondrial number and functionality.

Growing evidence indicates 3,5-diiodo-L-thyronine (T2) as a biologically active thyroid hormone derivative able to affect energy metabolism (Goglia, 2015 and references within). T2 increases resting metabolic rate, enhances lipid utilization as a fuel substrate, and prevents the occurrence of diet-induced obesity and associated diseases, including liver steatosis, hypertriglyceridemia, hypercholesterolemia (Lanni et al., 2005; de Lange et al., 2011), and insulin resistance (de Lange et al., 2011; Moreno et al., 2011). In SKM, T2 ameliorates the tissue's response to insulin that is impaired by a high fat diet (Moreno et al., 2011). Importantly, previous studies have shown that, contrary to T3, T2 does not induce thyrotoxicity or undesirable side effects at the cardiovascular level at the doses used (25 μg/100 g rat body weight, Lanni et al., 2005; de Lange et al., 2011).

The present mini review provides an overview of the involvement of SKM mitochondria in T3 and/or T2 effects exerted on modulation of SKM metabolism/plasticity. In particular, it focuses on signaling and biochemical pathways activated by the two iodothyronines in SKM, promoting variations in substrate metabolism, lipid handling, and thermogenesis at the mitochondrial level.

## Effect of thyroid hormones on SKM mitochondrial biogenesis

T3 influences mitochondrial activity and biogenesis by modulating, in a coordinate fashion, expression of proteins encoded by both the nuclear and mitochondrial genome.

### Nuclear events

T3 acts through nuclear receptors (TRs), namely TRα and TRβ, ligand-dependent transcription factors that are constitutively bound to thyroid hormone response elements (TREs). The binding of T3 to TRs leads to stimulation or inhibition of nuclear gene transcription (Brent, 2012). T3 regulates the transcription of a series of genes harboring TREs (direct T3 target genes), some of which serve as intermediate factors (e.g., transcriptional factors and coactivators) needed to regulate a second series of genes (indirect T3 target genes). In SKM, T3-modulated transcription is primarily mediated by the TRα1 isoform and involves a wide array of genes influencing SKM contractile and metabolic properties, as well as those coding components of the tricarboxylic acid cycle and mitochondrial respiratory chain (Wiesner et al., 1992; Short et al., 2001; Clement et al., 2002). In SKM, T3 also influences the transcription of genes controlling mRNA maturation and protein translation. Indeed, T3 up-regulates transcripts encoding ribunucleoproteins and splicing factors as well as ribosomal proteins and translation initiation factors (eIF1A, Clement et al., 2002).

T3 positively regulates the expression of intermediate factors, such as nuclear respiratory factors (NRF)-1 and -2, which enhance the expression of mitochondrial transcription factor-A, a nuclear-encoded transcription factor essential for replication, maintenance, and transcription of mitochondrial DNA. T3 also controls the expression of coactivator of peroxisome proliferator activated receptor γ (PPARγ) PGC-1α (Weitzel et al., 2001), a central regulator of mitochondrial gene expression and biogenesis (Puigserver, 2005). PGC-1α regulates gene expression through its interactions with DNA-bound transcription factors, including TR, PPAR, and NRF-1 (Knutti and Kralli, 2001, Figure 1).

**Figure 1:**
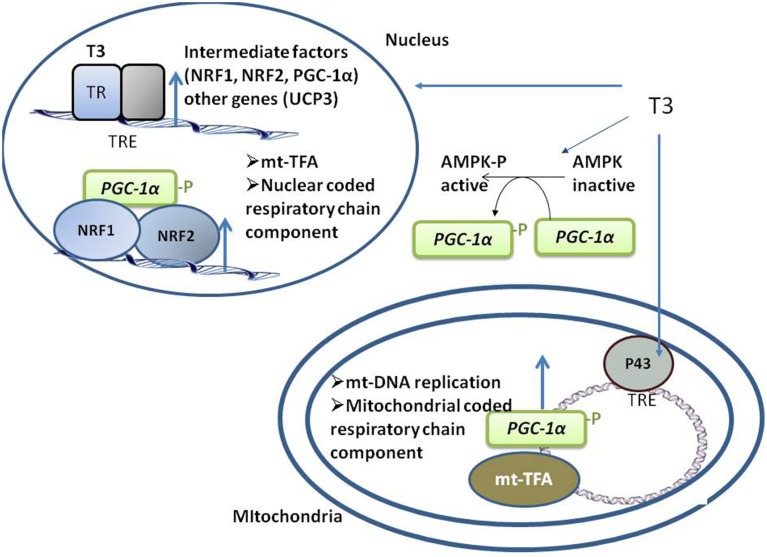
**Schematic representation of the coordinated events activated by T3 that take place in both nuclei and mitochondria, promoting mitochondrial biogenesis**. T3 directly activates the transcription of nuclear- and mitochondrial-genes coding for component of respiratory chain by binding to its nuclear TRs and mitochondrial p43. T3 also indirectly activates respiratory genes transcription by up-regulating the transcription of intermediate factors (such as NRF-1 and -2 and PGC1a). AMPK activation also mediates an indirect effect of T3 on mitochondrial biogenesis.

In rats, the effect induced by T3 on mitochondrial content and activity is amplified in slow oxidative compared to fast glycolytic muscles (Bahi et al., 2005). This could be explained by higher expression of TRα1 and PGC1α observed in slow oxidative muscle (Garnier et al., 2003; Bahi et al., 2005) associated with opposite regulation of TRα transcription by T3 in the two distinct muscle types (activation in slow oxidative and reduction in fast glycolytic muscle). Conversely, in humans, T3 does not influence NRF-1 or PGC1α levels in SKM (Barbe et al., 2001). Thus, the effect of T3 on SKM mitochondrial biogenesis seems to be species-specific and dependent on SKM metabolism.

In SKM, AMP-activated kinase (AMPK) regulates the expression of genes related to mitochondrial biogenesis, energy production, and oxidative protection. AMPK phosphorylates and activates PGC1α (Jäger et al., 2007; Cantó et al., 2009), and both chronic and acute administration of T3 to euthyroid (Irrcher et al., 2008) and hypothyroid rats (Branvold et al., 2008; de Lange et al., 2008) induces AMPK activation, a putative mediator of the effect of T3 on SKM mitochondrial biogenesis (Figure 1).

### Mitochondrial events

Mitochondrial gene expression is also directly activated by T3 through its binding to mitochondrial matrix-localized specific receptors (p43). p43 is a truncated form of TRα1 and is synthesized by the use of an internal initiation site of translation (Wrutniak et al., 1995; Wrutniak-Cabello et al., 2001). The T3–p43 complex binds to TREs of the mitochondrial genome and induces transcription (Casas et al., 1999) in parallel to the transcription of nuclear genes involved in oxidative phosphorylation, thus ensuring complementary signaling between nuclear and mitochondrial pathways (Figure 1). p43 regulates SKM phenotypes, contractile features, and metabolism. In mice, p43 deletion leads to muscle hypertrophy and a shift in the direction of more rapid muscle fiber types coordinated with a reduction in mitochondrial content (Pessemesse et al., 2012). p43 overexpression leads to muscle wasting with aging (Casas et al., 2009), suggesting a possible oxidative stress-associated toxic effect due to prolonged stimulation of mitochondrial activity, leading to a deficit of new skeletal muscle fiber replacement and differentiation over time.

P28 is another truncated form of TR-α1 that is localized in the inner mitochondrial membrane and shows a binding affinity for T3 higher than nuclear receptors. Despite it has been shown that p28 regulates mitochondrial functionality in fibroblast, its specific function has not been elucidated yet (Pessemesse et al., 2014).

### Effect of T2 on mitochondrial biogenesis

Studies supporting the possibility of T2's effects on mitochondrial biogenesis have been focused on liver and brown adipose tissue (de Lange et al., 2011; Lombardi et al., 2015). In SKM, it is possible that activation of AMPK by T2 (Lombardi et al., 2009a) could trigger transcriptional processes leading to mitochondrial biogenesis (see above). Direct evidence regarding the ability of T2 to influence mitochondrial biogenesis in SKM is currently lacking and this aspect needs further investigation.

Whether or not the effects of T2 are mediated by TRs is still under investigation. A recent study in mice showed that T2 evokes TR-mediated effects only when administered at very high doses (250 μg/100 g bw administered daily for 4 week, Jonas et al., 2015). When used at high doses an interaction of T2 with TRs could take place despite the much lower affinity of T2 for TRs when compared to T3 (Ball et al., 1997; Cioffi et al., 2010; de Lange et al., 2011; Mendoza et al., 2013). Interestingly, in a teleost fish species (Tilapia) T2 interacts with a TRβ receptor isoform and activates gene transcription *ex vivo* in a cell- and promoter-specific manner (Mendoza et al., 2013). Thus, further experiments are needed to elucidate whether and how T2 can modulate gene transcription.

## Thyroid hormones influence SKM mitochondrial functionality and thermogenesis

Mitochondrial functionality is profoundly affected by thyroid state. In SKM, in the transition between hypo- and hyper-thyroidism, a progressive increase in mitochondrial substrate oxidation is detected regardless of substrate [i.e., glycolytic- (Venditti et al., 2003, 2007) or lipid-associated substrates (Silvestri et al., 2005; Lombardi et al., 2012)].

### Mitochondrial uncoupling

Mitochondrial respiration is not fully coupled to ATP synthesis since part of the energy contained in the reduced substrate is lost as heat. Most of the uncoupling is due to a leak of protons across the mitochondrial inner membrane (proton-leak); a failure in proton pumping during electron transport (redox slip) also induces mitochondrial uncoupling. In SKM, proton-leak accounts for a significant portion of the cellular metabolic rate, either when muscle is at rest (Rolfe and Brand, 1996) or in the contracting state (Rolfe et al., 1999). Proton-leak is the sum of two processes: basal and inducible proton-leak (Brand and Esteves, 2005). Basal proton-leak is not acutely regulated. It depends on the fatty-acyl composition of the mitochondrial inner membrane and on the presence of adenine nucleotide translocase (ANT). Inducible proton-leak is acutely controlled by activation of specific proteins, with uncoupling protein (UCP3 in SKM) and ANT (ANT1 in SKM) playing a crucial role (Divakaruni and Brand, 2011).

### T3 induces SKM mitochondrial uncoupling

SKM mitochondrial uncoupling induced by T3 has been reported *in vivo* (Lebon et al., 2001) and *ex vivo* (Lanni et al., 1999; de Lange et al., 2001; Lombardi et al., 2002, 2012). Interestingly, despite uncoupling activation by T3, no variation or increase in SKM ATP levels takes place (Jucker et al., 2000; Lebon et al., 2001; Short et al., 2001). The uncoupling associated with T3-induced mitochondrial biogenesis could counteract possible ATP variations. In addition, an increase in the ability of SKM mitochondria to produce ATP could also take place, as already observed in liver (Harper and Brand, 1993; Nogueira et al., 2002).

The existence of a positive correlation between T3 and SKM mitochondrial proton-leak is also evident during aging. In fact, aging is associated with a decrease in circulating T3 that is evident in 24 month-old rats (Iossa et al., 2002; Silvestri et al., 2008; Valle et al., 2008); therefore, aging represents a condition of physiological hypothyroidism. Concomitantly, SKM mitochondria from old rats exhibited a significant decrease in proton-leak (Lombardi et al., 2009a; Crescenzo et al., 2014), suggesting that with increasing age, the efficiency of oxidative phosphorylation increases in SKM mitochondria. Similar results have been obtained *in vivo* in aged rat SKM, where a trend of higher coupling efficiency was found (Gouspillou et al., 2014). When mitochondria are more efficient, fewer substrates are oxidized to obtain ATP. Therefore, increased mitochondrial coupling in SKM could contribute to the decreased energy expenditure that characterizes the progression of aging and hypothyroidism since SKM energy metabolism accounts for about 30% of whole-body energy expenditure under resting conditions (Rolfe and Brown, 1997).

### Factors involved in T3 induced- SKM mitochondrial uncoupling

**Figure 2:**
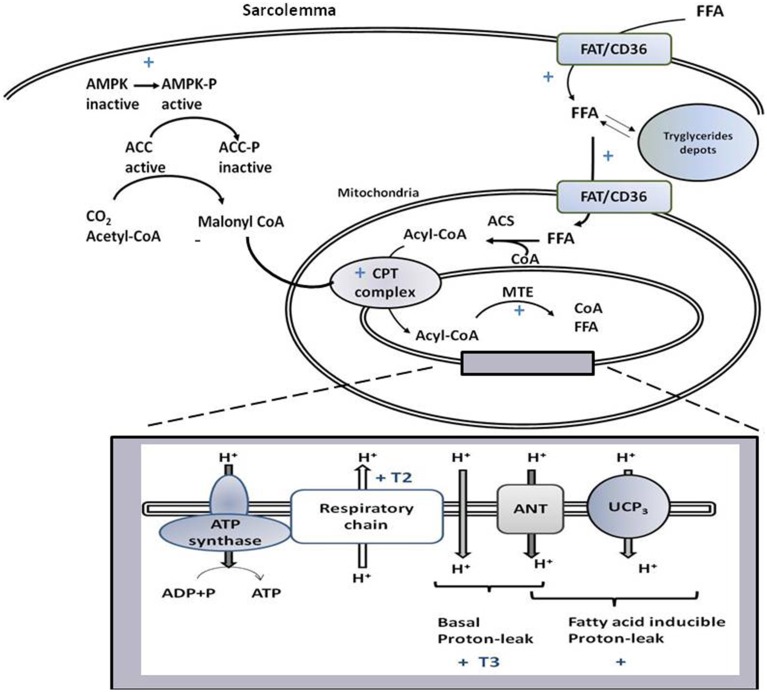
**Schematic representation of signaling and biochemical pathways activated by T3 and/or T2 in skeletal muscle that promote variations in substrate metabolism, lipid handling, and thermogenesis at the mitochondrial level**. If not accompanied by T3 or T2, the symbol + indicates that the pathway is stimulated by both iodothyronines. T3 and T2 activate processes leading to the import of FFA and their oxidation at the mitochondrial level, with FAT/CD36 playing a role. Through activation of AMPK-ACC signaling pathway, T2 and T3 relieves the inhibition of CPT1 by malonyl-CoA, and thus promote the entrance of fatty acids into mitochondria and their oxidation. The rise in MTE activity, would contribute to maintain a high level of fatty acid oxidation rate. The box represents processes, occurring at the level of mitochondrial inner membrane, underlying coupled and uncoupled respiration affected by T3 and T2.

T3 affects both basal (Lombardi et al., 2012) and inducible SKM proton-leak (Lanni et al., 1999; Silvestri et al., 2005; Lombardi et al., 2012), with UCP3 and ANT being involved in the effects on free fatty acid (FA)-inducible proton-leak (Figure 2). In the transition between hypo- and hyper-thyroidism, the contribution of ANT to FA-induced uncoupling becomes progressively more relevant despite there being no variation in ANT-1 mRNA levels detected (Dümmler et al., 1996; Lombardi et al., 2002). This could be attributed to the gradual increase in mitochondrial SKM FA levels (Lombardi et al., 2002), known activators of ANT-mediated uncoupling (Skulachev, 1991). Concerning UCP3, T3 increases its transcription (Lanni et al., 1999; Barbe et al., 2001), the effect being observed within 8 h of T3 administration to hypothyroid rats (de Lange et al., 2001, 2007). The mechanism of UCP3 promoter stimulation by T3 seems to be species-specific since it involves FA and their target receptors (PPARδ) in humans and rats but not mice (de Lange et al., 2007).

Besides regulating UCP3 expression, T3 also promotes UCP3-mediated uncoupling by synergistically stimulating biochemical pathways underlying activation of this protein (Silvestri et al., 2005). Indeed, T3 enhances reactive oxygen species formation (Venditti et al., 2003, 2007; Silvestri et al., 2005) and mitochondrial FA availability (Lombardi et al., 2002; Silvestri et al., 2005) that have been shown to act in combination to induce UCP3-mediated uncoupling (Echtay et al., 2002; Lombardi et al., 2008, 2010). A single administration of T3 to hypothyroid rats induces parallel increases in (i) whole animal resting metabolic rate, (ii) SKM mitochondrial UCP3 content, and (iii) SKM mitochondrial uncoupling, thus indicating the importance of UCP3 in the regulation of rat resting metabolic rate by T3 (de Lange et al., 2001; Flandin et al., 2009). UCP3 is also involved in mitigation of reactive oxygen species production (Brand and Esteves, 2005) and counteracting lipotoxicity induced by accumulation of FA and lipid hydroperoxides in the mitochondrial matrix (Goglia and Skulachev, 2005; Schrauwen et al., 2006; Lombardi et al., 2010). Thus, the upregulation of UCP3 by T3 would alleviate mitochondrial damage resulting from chronic mitochondrial activation associated with hyperthyroidism.

### 3,5-T2 affects mitochondrial oxidative phosphorylation in SKM

The administration of T2 to hypothyroid rats rapidly enhances both coupled and uncoupled respiration with mechanisms that are independent of de novo transcription and translation (Lombardi et al., 2007). Indeed, T2 promotes activation of the kinetics of the reactions involved in the oxidation of substrates (among these respiratory chain), while not primarily influencing reactions involved in the synthesis and export of ATP (Lombardi et al., 2007, Figure 2).

The mechanism by which T2 affects uncoupled mitochondrial respiration mainly involves proton leak, since T2 does not affect redox slip nor induce any significant change in the overall respiratory chain H^+^/O ratio (Lombardi et al., 2007). Contrary to what is observed for T3, T2 does not activate basal proton-leak, rather its effect is totally dependent on FA presence (Lombardi et al., 2009a, 2012). Although, it is clear that T2 promotes FA-inducible proton-leak, the molecular component involved have not been individuated yet (Figure 2).

## Thyroid hormones influence SKM mitochondrial lipid handling

Alterations in thyroid state are associated with changes in energy demand, with SKM adapting its metabolism by modulating substrate utilization. In the hypothyroid state, SKM enhances FA import into myocytes, a process associated with a decrease in the ability of mitochondria to use FA as fuel and enhancement of oxidative phosphorylation efficiency. Thus, the imbalance between FA supply and oxidation leads to accumulation of intra-myocyte triglycerides (Lombardi et al., 2012). On the other hand, in the hyperthyroid condition, an increase in FA uptake into myocytes is associated with a rise in FA oxidation, which becomes less efficient because of proton-leak activation. Consequently, FAs are not deposited as triglycerides (Lombardi et al., 2012, Figure 2).

### T3 affects mitochondrial fatty acid oxidation

More processes are crucial for SKM mitochondrial FA oxidation, which include FA availability to mitochondria, import of acyl-CoA into the mitochondrion, mitochondrial oxidative capacity, and feedback inhibition by intermediates present in the FA oxidation pathway. T3 influences all the cited processes. Indeed, it promotes mitochondrial localization of FAT/CD36 (Lombardi et al., 2012), known to increase FA supply to the mitochondria (Holloway et al., 2009). This event is coordinated with the import of acyl-CoA into the mitochondria, obtained by activation of the carnitine-palmitoyl-transferase (CPT) complex (considered a rate-limiting step for FA uptake into mitochondrion). T3 modulates transcription of CPT complex components (e.g., CPT-1 and -2, Silvestri et al., 2005). In addition, in SKM, T3 promotes AMPK activation and inhibition of its downstream target, acetyl-CoA carboxylase (ACC, Branvold et al., 2008; de Lange et al., 2008; Irrcher et al., 2008). ACC inhibition leads to a reduction in malonyl-CoA levels that inhibits CPT-1 activity. Thus, the activation of AMPK-ACC-malonyl-CoA signaling leads to sequential enhancement of CPT-1 activity, mitochondrial acyl-CoA uptake, and oxidation (de Lange et al., 2008, Figure 2).

### Mitochondrial lipid handling and uncoupling: interrelated role in mediating the effect of T3 on SKM mitochondria

Inside mitochondria, a rise in NADH/NAD^+^ and CoA-SH/acetyl-CoA ratios, as well as accumulation of β-oxidation intermediate metabolites, can cause feedback inhibition of the β-oxidation pathway (Koves et al., 2008). The T3-induced uncoupling effect contributes to maintaining the above ratios at low levels and thus, functioning to sustain an elevated mitochondrial FA oxidation rate. Furthermore, the activation of SKM intra-mitochondrial thioesterase (MTE; catalyzes cleavage of acyl-CoA to CoA and free FA) by T3 (Silvestri et al., 2005) contributes to sustaining a high FA oxidation rate, since it maintains a high CoA/acetyl-CoA ratio and supplies CoA, whose pool is limited, for β-oxidation. Intra-mitochondrial production of FA, catalyzed by MTE, could play a role in FA-induced proton leak activated by T3. Thus, an interlink between lipid-handling and mitochondrial uncoupling coexists: the activation of uncoupling could facilitate the FA oxidation rate and, at the same time, the increase availability of FA to mitochondria, associated with lipid handling, would promote FA-induced mitochondrial uncoupling.

### T2 affects SKM fatty acid oxidation

The rapid stimulatory effect of T2 on mitochondrial respiration seems to be specific to FA metabolism, since T2 does not influence mitochondrial ability to use pyruvate as a substrate (Lombardi et al., 2009a). T2 has an effect similar to that induced by T3 in increasing SKM FA uptake and channeling FAs to mitochondria and increasing their oxidation. Indeed, T2 and T3 activate translocation of FAT/CD36 from cellular depots to the sarcolemma and mitochondria, each in a very rapid fashion. In this aspect, the two iodothyronines seem to mimic the effect of physical exercise, which influences FAT/CD36-mediated transport of lipids across the sarcolemmal membrane and into the mitochondria (Holloway et al., 2009).

Although both T2 and T3 increase the SKM mitochondrial FA oxidation rate in hypothyroid rats, the onset of CPT and mitochondrial respiratory pathway activation differ since the two processes were already activated 1 h after T2 administration, whereas T3 was ineffective at that time point (Lombardi et al., 2012). Within 1 h, T2 rapidly activated the AMPK-CPT-malonyl-CoA signaling pathway that leads to enhancement of FA uptake in mitochondria via increased CPT-1 activity (Figure 2). Rapid activation of MTE-1 and proton-leak by T2 would contribute to maintaining high FA oxidation rates (Lombardi et al., 2009b).

## Conclusions

SKM mitochondrial physiology is profoundly affected by the thyroid state and underlies a significant part of the metabolic effects induced by T3. The recent discovery of T2 as a metabolically active thyroid hormone derivatives indicates that thyroid physiology is continually evolving. These novel aspects of thyroid physiology could reveal new perspectives for understanding the contribution of SKM mitochondria to different thyroid states.

### Conflict of interest statement

The authors declare that the research was conducted in the absence of any commercial or financial relationships that could be construed as a potential conflict of interest.
